# A note on integrated local energy decay estimates for spherically symmetric black hole spacetimes

**DOI:** 10.1007/s00208-026-03482-w

**Published:** 2026-04-30

**Authors:** Gustav Holzegel, Georgios Mavrogiannis, Renato Velozo Ruiz

**Affiliations:** 1 Department of Mathematics, Imperial College London, South Kensington Campus, London, SW7 2AZ UK; 2 Mathematisches Institut, Universität Münster, Einsteinstrasse 62, 48149 Münster, Germany; 3 Department of Mathematics, Rutgers University, New Brunswick, NJ 08903 USA; 4 Department of Mathematics, University of Toronto, 40 St. George Street, Toronto, ON Canada

**Keywords:** 58J45, 35L10, 83C05, 83C57

## Abstract

We present short proofs of integrated local energy decay estimates on Schwarzschild, extremal Reissner–Nordström, and Schwarzschild–de Sitter spacetimes. The proofs employ novel global physical space multipliers, which, besides their remarkable simplicity (a) are directly derivable from the geodesic flow, (b) do not require decomposition into spherical harmonics, and (c) whose boundary terms can be controlled by the conserved *T*-energy alone. We also elaborate on the intimate connection between the multipliers of the present paper and the globally good commutators introduced in Holzegel and Kauffman (J Hyperbolic Differ Equ 20(4):825–834, 2023) and Mavrogiannis (Ann Henri Poincaré 24(9):3113–3152, 2023).

## Introduction

Integrated local energy decay estimates for hyperbolic equations on the exterior of black hole backgrounds have been studied intensely in the past 2 decades, most often in connection with the stability problem for black holes [1, 6, 7, 12, 14, 33, 35]. The importance of such estimates lies in their global character, as they necessarily capture the geometry of the entire spacetime, including the well-known phenomenon of trapped geodesics and its associated degeneration in the estimate. Moreover, once such an estimate has been established, it can be combined with well-known estimates near the horizon (going back to [12]) and near the asymptotically flat end [13] to prove inverse polynomial decay estimates and more refined decay statements including, with additional work, sharp decay rates [2–4, 22].

In this paper, we revisit some of the simplest settings where an integrated local energy decay estimate can be proven: The covariant wave equation $\Box _{g} \psi =0$ on $(\mathcal {M},g)$ the exterior of the Schwarzschild, the extremal Reissner–Nordström, and the Schwarzschild–de Sitter black hole geometry. Note that being static, these examples admit a coercive energy conservation law from the timelike Killing field. Our main motivation here is not so much to produce new results, although some of the estimates we state are new (see the comments after Theorems 2 and 4 in Sect. 3), but rather to

1. Streamline existing proofs by providing simple global multipliers leading to very short proofs.
2. Derive the exact form of these multipliers from global considerations regarding geodesic flow.

For the Schwarzschild geometry, the proof of such estimates goes back to [7, 12], who provided the first, rather elaborate, constructions of multipliers leading to integrated local energy decay estimates. However, in both works, the multipliers depended on the angular momentum number $\ell$ (of a decomposition of the solution into spherical harmonics), which complicated direct non-linear applications. This drawback was resolved in [10] at the cost of commuting with angular momentum operators, i.e. invoking a higher order energy. Finally, [28] provided a construction of a single multiplier that worked for all frequencies without any commutation. Their construction relied on the redshift vector field of [12]: a small amount of it had to be added in the construction to compensate for terms of the wrong sign near the horizon. In particular, the estimate of [28] does not provide an integrated local energy that can be controlled by the *T*-energy arising from the Killing field. In contrast, our Theorem 1 below controls an integrated decay norm in terms of the uncommuted initial *T*-energy alone. Remarkably, its proof only invokes multiplying the covariant wave Eq. (2.3) by $X\psi$ where *X* is (expressed in standard Schwarzschild coordinates)

1.1 $$\begin{aligned} X&= \left( 1-\frac{2M}{r}\right) f(r) \partial _r + 2\left( 1-\frac{2M}{r}\right) (\partial _r f) (r), \ \ \ \text {where} \nonumber \\ f(r)&= \left( 1-\frac{3M}{r}\right) \sqrt{1+\frac{6M}{r}}. \end{aligned}$$

While the degenerations at the horizon and the trapped set in (1.1) are well-known, the factor of $\sqrt{1+\frac{6M}{r}}$ may look rather artificial. However, the exact form of *f* is obtained when writing down the radial momentum $p^r$ (as a function of *r*) corresponding to a future-trapped null geodesic, i.e. a null geodesic approaching $r=3M$ asymptotically. We note that the form of *f* also appeared in the commutator vector fields introduced in [25, 29]. We elaborate on this connection in Sect. 5.

The unifying principle of “deriving” the *global* form of the multiplier from the parametrisation of trapped geodesics in phase space is at the heart of this paper. It is remarkable and still slightly mysterious to us that the multipliers thus obtained provide direct and simple proofs of integrated local energy decay estimates in many classical cases: The Schwarzschild geometry in higher dimensions (here our Theorem 2, which concerns $n=4+1$ dimensions, considerably simplifies the constructions in [27, 32]), the extremal Reissner–Nordström geometry (here our Theorem 3 simplifies the construction in [6]) and the Schwarzschild–de Sitter geometry (our Theorem 4, which concerns the conformal wave equation).

We note that in the high frequency limit and locally near the trapped set, the geometric construction of multipliers in the present paper, connects with previous work on resolvent estimates for trapped sets, see for example [8, 19, 37, 17, Proposition 7.5]. To obtain these results, the aforementioned authors use the Hamiltonian flow, with appropriate test functions, and obtain coercive frequency localized estimates in a neighbourhood of the (normally hyperbolic) trapping. In comparison, the multipliers we derive and apply in the present paper generate estimates that are global both in spacetime and frequencies.

It is of course a natural question whether the procedure to construct multipliers outlined above leads to novel microlocal multipliers and a simplification of [14] in the Kerr case. We discuss this briefly in Sect. 6. However, in order not to blur the simplicity of the construction in the basic examples, we postpone a detailed investigation of this to the future.

## Preliminaries

### The spacetimes

We study the (static, spherically symmetric) black hole exteriors corresponding to the Schwarzschild, the extremal Reissner–Nordström and the Schwarzschild–de Sitter metric. We let $(\mathcal {M},g)$ be an *n*-dimensional Lorentzian manifold $(\mathcal {M},g)$ with $\mathcal {M}=\mathbb {R}_t \times (\rho _1,\rho _2)_r \times \mathbb {S}^{n-2}$ and

2.1 $$\begin{aligned} g= -\xi (r) dt\otimes dt +(\xi (r))^{-1}dr\otimes dr +r^2 d\sigma _{\mathbb {S}^{n-2}}, \end{aligned}$$

where $\xi : (\rho _1,\rho _2) \rightarrow \mathbb {R}^+$ is smooth, and $d\sigma _{\mathbb {S}^{n-2}}$ denotes the round metric on the unit sphere $\mathbb {S}^{n-2}$.

Specifically, given $M>0$, we define

1. $\textrm{Schw}^{1+3}:=\mathbb {R}_t\times (2M,\infty )_r\times \mathbb {S}^2$ with $\xi (r) = 1-\frac{2M}{r}$ and $n=4$ in (2.1),
2. $\mathrm {Schw^{1+4}}:=\mathbb {R}_t\times (\sqrt{2M},\infty )_r\times \mathbb {S}^3$ with $\xi (r)=1-\frac{{2M}}{r^{2}}$ and $n=5$ in (2.1),
3. $\textrm{ERN}^{1+3}:=\mathbb {R}_t\times (M,\infty )_r\times \mathbb {S}^2$ with $\xi (r)=(1-\frac{M}{r})^2$ and $n=4$ in (2.1).

Given also $L>0$ and $M>0$ that satisfy the subextremality condition $0<\frac{M^2}{L^2}<\frac{1}{27}$ and letting $\bar{r}_+>r_+>0$ denote the roots of $\xi (r)=1- \frac{2M}{r}-\frac{r^2}{L^2}$:

(4) $\mathrm {SchwdS^{1+3}}:=\mathbb {R}_t\times (r_+,\bar{r}_+)_r\times \mathbb {S}^2$ with $\xi (r)=1- \frac{2M}{r}-\frac{r^2}{L^2}$ and $n=4$ in (2.1).

We will often employ the well-known tortoise coordinate $r^\star \in (-\infty ,\infty )$ defined through $\frac{dr^\star }{dr} = \frac{1}{\xi (r)}$.

Finally, note that the natural spacetime volume form associated with the metrics (2.1) can be written as $dvol_{\mathcal {M}} = \xi r^{n-2} dt\wedge dr^\star \wedge dvol_{\mathbb {S}^{n-2}}$, where $dvol_{\mathbb {S}^{n-2}}$ denotes the standard volume form on $\mathbb {S}^{n-2}$.

### The spacetime foliations

For each of the spacetimes of Sect. 2.1 above we consider in addition a foliation by spherically symmetric, smooth spacelike slices $\Sigma _\tau$, which are defined as the push-forward $\Sigma _\tau = \varphi _{\tau }(\Sigma _0)$ associated with the integral curves of the Killing vector field $\partial _t$, where for (1)–(3) the leaf $\Sigma _0$ is chosen to connect the future event horizon with future null infinity, and for (4) the leaf $\Sigma _0$ is chosen to connect the future event horizon and the future cosmological horizon. The precise form of the slices is inessential in what follows. (In the cases (1)–(3) all of our arguments would equally work for slices ending at spatial infinity.) However, to make things more explicit, let us define the $\Sigma _\tau$ to be slices of constant $\tau$, where $\tau :=t+h(r)$ is a function on $\mathcal {M}$ defined as follows: We fix $0<\delta <1$ and let $h: (\rho _1,\rho _2) \rightarrow \mathbb {R}$ be a smooth function, which satisfies $h^\prime (r)= +(1-\xi ) \frac{1}{\xi }$ in a neighbourhood of $\rho _1$ and $h^\prime (r) = -(1-(\frac{\rho _1}{r})^{1+\delta })\frac{1}{\xi }$ in a neighbourhood of $\rho _2$ and smoothly interpolates inbetween such that $|h^\prime (r)|<\frac{1}{\xi }$ (making the hypersurface globally timelike). Note that the normal $n_{\Sigma _\tau }$ to $\Sigma _\tau$ is then proportional to $\frac{1}{\xi } \partial _t - (1-\xi )\frac{1}{\xi } \partial _{r^\star }$ in the region near $\rho _1$, and proportional to $\frac{1}{\xi } \partial _t + (1-(\frac{\rho _1}{r})^{1+\delta })\frac{1}{\xi } \partial _{r^\star }$ in the region near $\rho _2$.

Finally, the region between two slices $\Sigma _{\tau _1}$, $\Sigma _{\tau _2}$ will be denoted $\mathcal {M}\left( \tau _1,\tau _2\right)$.

### The covariant wave equation

We will consider smooth solutions to the covariant wave equation

2.2 $$\begin{aligned} \Box _g \phi - \mu \phi = 0 \, \end{aligned}$$

in the following four cases (recall the spacetimes defined in Sect. 2.1):

- $\mu = 0$ in (2.2) and $(\mathcal {M},g)$ being one of the spacetimes $\textrm{Schw}^{1+3}$, $\mathrm {Schw^{1+4}}$ or $\textrm{ERN}^{1+3}$.
- $\mu =\frac{2}{L^2}$ in (2.2) and $(\mathcal {M},g)$ being the spacetime $\mathrm {SchwdS^{1+3}}$.

Note that all cases arise from the conformal wave equation in dimension *n*, $\Box _g \phi - \frac{n-2}{4(n-1)}R_g \phi =0$, where $R_g$ is the scalar curvature of the metric *g*. (Indeed, (1)–(3) are Ricci-flat and (4) satisfies $Ric_g=\frac{3}{L^2}g$.) The fundamental solution of the conformal wave equation is supported on the lightcones of *g*, hence all cases above correspond to the propagation of massless particles or waves in their respective geometry.

Setting $\psi = \phi r^{\frac{n-2}{2}}$, we can write (2.2) as

2.3 $$\begin{aligned}&-\partial _t^2 \psi + \partial _{r^\star }^2 \psi + \frac{\xi (r)}{r^2} \Delta _{\mathbb {S}^{n-2}} \psi -V(r) \psi - \mu \xi (r) \psi = 0 \ , \ \ \ \text {where}\nonumber \\&V(r) = r^{-\frac{n-2}{2}} \left( r^{\frac{n-2}{2}}\right) ^{\prime \prime }. \end{aligned}$$

In the above, a prime denotes differentiation with respect to $r^\star$ and $\Delta _{\mathbb {S}^{n-2}}$ is the spherical Laplacian on the unit sphere. It will often be algebraically simpler to work with (2.3) directly instead of (2.2). Note that in all four cases considered there is a $c>0$ depending only on the black hole parameters such that

2.4 $$\begin{aligned} V+\mu \xi \ge \frac{c}{r^3}\xi \ \ \ \text {holds for } r \in (\rho _1,\rho _2). \end{aligned}$$

### The basic energy currents

Using notation of [16, Section 3.3], we define from a tuple $(X,w,q,\varpi )$ consisting of a vectorfield *X*, a function *w*, a one-form *q* and an $(n-2)$-form $\varpi$ on $\mathcal {M}$, the currents

2.5 $$\begin{aligned} J_\mu ^{X, w, q, \varpi } [\phi ]&:= T_{\mu \nu } [\phi ] X^\nu + w \phi \partial _\mu \phi + q_\mu \phi ^2 + \star d(\phi ^2 \varpi )_\mu \, , \end{aligned}$$

2.6 $$\begin{aligned} K^{X,w,q} [\phi ]&:= T^{\mu \nu } \pi ^X_{\mu \nu } + \nabla ^\mu w \phi \nabla _\mu \phi + w \left( \nabla ^\mu \phi \nabla _\mu \phi + \mu \phi ^2\right) \nonumber \\&\quad + \nabla ^\mu q_\mu \phi ^2 + 2 \phi q_\mu g^{\mu \nu } \partial _\nu \phi \, , \end{aligned}$$

where

2.7 $$\begin{aligned} T_{\mu \nu }[\phi ]&= \partial _\mu \phi \partial _\nu \phi - \frac{1}{2} g_{\mu \nu } \left( g^{\alpha \beta } \partial _\alpha \phi \partial _\beta \phi + \mu \phi ^2 \right) \, , \nonumber \\ \pi _{\mu \nu }^X&= \frac{1}{2} (\mathcal {L}_X g)_{\mu \nu } = \frac{1}{2} \left( \nabla _\mu X_\nu +\nabla _\nu X_\mu \right) . \end{aligned}$$

The $\star$ appearing in (2.5) is the Hodge star operator $\star : \Lambda ^k \mathcal {M} \rightarrow \Lambda ^{n-k} \mathcal {M}$ defined for $k=0,1,\ldots ,n$ by $\alpha \wedge \star \beta = g(\alpha , \beta ) dvol_{\mathcal {M}}$ for $\alpha , \beta \in \Lambda ^k \mathcal {M}$ and satisfying $(\star \star \alpha )=-(-1)^{k(n-k)}\alpha$ for $\alpha \in \Lambda ^k \mathcal {M}$.

One easily checks that for $\phi$ satisfying (2.2), the above currents are related by the divergence identity

2.8 $$\begin{aligned} \nabla ^\mu J_\mu ^{X, w, q, \varpi } [\phi ] = K^{X,w,q} [\phi ] \, . \end{aligned}$$

Note that the term $\star d(\phi ^2 \varpi )_\mu$ in (2.5) denotes the components of a 1-form which is manifestly divergence free in view of $d^2=0$ and the formula for the divergence of a 1-form $\xi \in \Lambda ^1 \mathcal {M}$, $div \xi = \star d \star \xi$.

Below we will consider two currents $J^{(1)}=J^{X_1,w_1,q_1,\varpi _1}[\phi ]$ and $J^{(2)}=J^{X_2,w_2,q_2,\varpi _2}[\phi ]$.

The current $J^{(1)}$ arises from the static Killing field $T=\partial _t$ and is defined by

2.9 $$\begin{aligned} X_1 = \partial _t, \quad w_1= 0, \quad q_1= 0, \quad \varpi _1 = \left( -1\right) ^{n+1} \frac{n-2}{4} \frac{\xi }{r} r^{n-2} d\text {vol}_{\mathbb {S}^{n-2}} \, . \end{aligned}$$

Clearly $K^{X_1,w_1,q_1}[\phi ]=0$ and one computes the components (expressed in terms of $\psi =r^{\frac{n-2}{2}} \phi$)

2.10 $$\begin{aligned} J^{(1)}_t&= \frac{1}{2} \frac{1}{r^{n-2}} \left( (\partial _t \psi )^2 + (\partial _{r^\star } \psi )^2 +\frac{\xi (r)}{r^2} |\nabla _{\mathbb {S}^{n-2}} \psi |^2 +\left( V(r) + \mu \xi (r) \right) \psi ^2\right) \, , \nonumber \\ J^{(1)}_{r^\star }&= \frac{1}{r^{n-2}} \left( \partial _t \psi \partial _{r^\star } \psi \right) \, , \nonumber \\ J^{(1)}_A&= \frac{1}{r^{n-2}} \left( \partial _t \psi \partial _{A} \psi \right) . \end{aligned}$$

To obtain (2.10) note that $\star dt = -r^{n-2} dr^\star \wedge dvol_{\mathbb {S}^{n-2}}$ and $dt =(-1)^{n-1} r^{n-2} \star (dr^\star \wedge dvol_{\mathbb {S}^{n-2}})$, hence

$$\begin{aligned} \star d(\phi ^2 \varpi _1)_t&= \frac{1}{4}(-1)^{n+1} \star d(\phi ^2 (r^{n-2})^\prime dvol_{\mathbb {S}^{n-2}})_t \nonumber \\  &= \frac{1}{4} (-1)^{n+1}\left( 2\phi \phi ^\prime (r^{n-2})^\prime + \phi ^2 (r^{n-2})^{\prime \prime }) \right) (\star (dr^\star \wedge dvol_{\mathbb {S}^{n-2}}))_t \nonumber \\&= \frac{1}{4} \frac{1}{r^{n-2}} \left( 2\phi \phi ^\prime (r^{n-2})^\prime + \phi ^2 (r^{n-2})^{\prime \prime }) \right) ,\nonumber \end{aligned}$$

from which it is easy to verify that

$$\begin{aligned}&\frac{1}{2}(\phi ^\prime )^2 + \frac{1}{4} \frac{1}{r^{n-2}} \left( 2\phi \phi ^\prime (r^{n-2})^\prime + \phi ^2 (r^{n-2})^{\prime \prime }) \right) \nonumber \\  &\quad = \frac{1}{2} \frac{1}{r^{n-2}} \left[ (r^{\frac{n-2}{2}} \phi )^\prime \right] ^2 + \frac{1}{2}\frac{V(r)}{r^{n-2}} (r^{\frac{n-2}{2}} \phi )^2 \, . \end{aligned}$$

#### Remark 2.1

The addition of the non-trivial $(n-2)$-form in (2.9) therefore produces the additional coercive zeroth order term $V\psi ^2$ in the current $J^{(1)}_t$ when written in terms of $\psi$ instead of $\phi$, which is convenient in the analysis. For $\varpi _1=0$ one would obtain the usual $J^{(1)}_t = \frac{1}{2} \left( (\partial _t \phi )^2 + (\partial _{r^\star } \phi )^2 +\frac{\xi (r)}{r^2} |\nabla _{\mathbb {S}^{n-2}} \phi |^2 +\mu \xi (r)\phi ^2\right)$.

The current $J^{(2)}=J^{X_2,w_2,q_2,\varpi _2}$ is the so-called Morawetz current, defined by

2.11 $$\begin{aligned} X_2&= f\partial _{r^\star } ,\quad w_2=\frac{n-2}{2} \frac{f}{r}\xi + \frac{1}{2}f^\prime ,\nonumber \\ (q_2)_\mu&= -\frac{1}{2} \partial _\mu w_2 + \frac{n-2}{2} \frac{f^\prime }{r} \partial _\mu r , \quad \varpi _2 = 0 \, , \end{aligned}$$

for a smooth radial function $f: (\rho _1,\rho _2) \rightarrow \mathbb {R}$ to be determined in (2.23). We compute its components

2.12 $$\begin{aligned} J^{(2)}_t&= \frac{1}{r^{n-2}} \left( f \partial _t \psi \partial _{r^\star } \psi + \frac{1}{2} f^\prime \partial _t \psi \cdot \psi \right) \, , \nonumber \\ J^{(2)}_{r^\star }&= \frac{1}{2} \frac{1}{r^{n-2}} \left( f(\partial _{r^\star } \psi )^2 + f(\partial _{t} \psi )^2 - f\frac{\xi (r)}{r^2} |\nabla _{\mathbb {S}^{n-2}} \psi |^2 \right. \nonumber \\&\quad \left. -(V+\mu \xi (r)) f \psi ^2 + f^\prime \partial _{r^\star } \psi \cdot \psi -\frac{f^{\prime \prime } \psi ^2}{2}\right) \, , \nonumber \\ J^{(2)}_A&= \frac{1}{r^{n-2}} \left( \partial _{r^\star } \psi \partial _{A} \psi \right) , \end{aligned}$$

and the spacetime term $K^{(2)}=K^{X_2,w_2,q_2}[\phi ]$:

2.13 $$\begin{aligned} K^{(2)}= \frac{1}{r^{n-2}\xi } \left[ f^\prime (\partial _{r^\star } \psi )^2 - \frac{f}{2} \left( \frac{\xi (r)}{r^2}\right) ^\prime |\nabla _{\mathbb {S}^{n-2}} \psi |^2- \left( \frac{1}{4} f^{\prime \prime \prime } + \frac{1}{2}(V+\mu \xi )^\prime f\right) \psi ^2 \right] . \end{aligned}$$

We finally remark that the divergence identities associated with $J^{(1)}$ and $J^{(2)}$ can also be obtained directly “by hand” if ones multiplies (2.3) by $\partial _t \psi$ and by $-f^\prime \psi -2 f\psi ^\prime$ respectively.

### The basic energy

We define the basic energy on the spacelike slices $\Sigma _\tau$ introduced in Sect. 2.2,

2.14 $$\begin{aligned} \mathbb {E}^T[\psi ](\tau ):=\int _{\Sigma _\tau } J_\mu ^{(1)} n^\mu _{\Sigma }. \end{aligned}$$

Using (2.4) one finds that $\mathbb {E}^T[\psi ](\tau )$ is coercive in each of the four cases considered. More precisely, using the expressions for the normals in Sect. 2.2 one finds in cases (1)–(3):

2.15 $$\begin{aligned} \mathbb {E}^T[\psi ](\tau )&\sim \int _{\Sigma _{\tau }} dr d\text {vol}_{\mathbb {S}^{n-2}} \left[ (\partial _t \psi + \partial _{r^\star }\psi )^2 + \frac{ (\partial _t \psi - \partial _{r^\star }\psi )^2}{\xi (r) r^{1+\delta }}\right. \nonumber \\&\quad \left. +\frac{\xi (r)}{r^2} |\nabla _{\mathbb {S}^{n-2}} \psi |^2 +\frac{\xi }{r^3} \psi ^2 \right] , \end{aligned}$$

where $0<\delta <1$ is the parameter appearing in the definition of the $\Sigma _\tau$ in Sect. 2.2 and $\sim$ involves only constants depending on the black hole parameters. In case (4) we also have (2.15) provided we replace $(\partial _t \psi + \partial _{r^\star }\psi )^2 + \frac{ (\partial _t \psi - \partial _{r^\star }\psi )^2}{\xi (r) r^{1+\delta }}$ in the integrand by $\frac{ (\partial _t \psi + \partial _{r^\star }\psi )^2 }{\bar{r}_+-r} + \frac{ (\partial _t \psi - \partial _{r^\star }\psi )^2}{r-r_+}$.

Applying the divergence identity (2.8) for the current $J^{(1)}_\mu$ yields for any $\tau _2 \ge \tau _1$ the estimate

2.16 $$\begin{aligned} \mathbb {E}^T[\psi ](\tau _2) \le \mathbb {E}^T[\psi ](\tau _1) . \end{aligned}$$

### Deriving the multipliers from the geodesic flow

In this subsection, we derive a suitable ansatz for the radial function in the current we will use in the proofs of our results.

#### The geodesic flow and the radial geodesic motion

Let $(\mathcal {M},g)$ be one of the spacetimes in (1)–(4). The dynamics of free falling massless particles on $\mathcal {M}$ takes place on the set $\mathcal {P}\subset T\mathcal {M}$ defined by

2.17 $$\begin{aligned} \mathcal {P}:=\big \{(x,p)\in T\mathcal {M}: g_x(p,p) =0, \text { where } p \text { is future directed}\big \}\, . \end{aligned}$$

Here, $(p^t, p^r, p^A)$ are standard dual momentum coordinates on the fibers of $T\mathcal {M}$. We consider the geodesic flow defined by the equations

$$\begin{aligned} \frac{dx^{\alpha }}{ds}=p^{\alpha } ,\quad \frac{dp^{\alpha }}{ds}=-\Gamma ^{\alpha }_{\beta \gamma }p^{\beta }p^{\gamma }, \end{aligned}$$

where $\Gamma ^{\alpha }_{\beta \gamma }$ are the Christoffel symbols of *g* with respect to the chart coordinate system $(t,r,\theta ^{A})$. The conserved quantities associated with staticity and spherical symmetry of the metric make the geodesic flow a *completely integrable* Hamiltonian flow in the sense of [38, Section V.10]. These conserved quantities can be used to reduce the radial geodesic motion to an ODE involving only the (conserved) *particle energy* *E* and the (conserved) *total angular momentum* *l* defined by

$$\begin{aligned} E:=\xi \cdot p^t, \quad l:=r\sqrt{g_{AB}p^A p^B} . \end{aligned}$$

Indeed, by (2.17), the particle energy *E* can be written in terms the coordinates $r$ and $p^r$ by

2.18 $$\begin{aligned} E^2=(p^r)^2+\mathcal {V}_l(r),\quad \mathcal {V}_l(r):=\dfrac{l^2}{r^2}\xi . \end{aligned}$$

We refer to $\mathcal {V}_l(r)$ as the radial potential of the geodesic flow. Differentiating (2.18) along the flow, we obtain the radial geodesic equation

2.19 $$\begin{aligned} \frac{d^2 r}{ds^2}=\partial _r \mathcal {V}_l(r), \end{aligned}$$

which can also be written as a system of first order ODEs as

2.20 $$\begin{aligned} \frac{d}{ds}\begin{pmatrix} r \\ p^r \end{pmatrix} =F_{l} \begin{pmatrix} r \\ p^r \end{pmatrix} ,\quad F_{l} \begin{pmatrix} r \\ p^r \end{pmatrix} :=\begin{pmatrix} p^r \\ \partial _r \mathcal {V}_l(r) \end{pmatrix}, \end{aligned}$$

where $F_l$ is a fixed map defined on $U=(2\,M,+\infty )_r\times \mathbb {R}_{p^r}$. The system of ODEs (2.20) defines the radial geodesic flow $(r(s),p^r(s))$ for fixed angular momentum $l$.

#### Trapped null geodesics

For the spacetimes (1)–(4), the potential $\mathcal {V}_l$ has a unique critical point at $r=r_{\textrm{trap}}$. This critical point is a local maximum of $\mathcal {V}_l$. From here, it follows that $(r_{\textrm{trap}},0)$ is a hyperbolic fixed point of the radial flow $(r(s),p^r(s))$ for fixed $l$.^1^ This fixed point corresponds to null geodesics on the photon sphere $\{r=r_{\textrm{trap}}\}$. Recall that *trapped orbits* are geodesics in the so-called *trapped set*, given by

2.21 $$\begin{aligned} \Gamma =\big \{ (x,p)\in \mathcal {P}: r=r_{\textrm{trap}}, \quad p^r=0\big \}. \end{aligned}$$

More generally, we say that geodesics are *future* or *past trapped*, if $\lim _{s\rightarrow \infty }(r(s), p^r(s))=(r_{\textrm{trap}},0)$ or $\lim _{s\rightarrow -\infty }(r(s),p^r(s))=(r_{\textrm{trap}},0)$, respectively. Along future and past trapped orbits, the conserved quantities satisfy $\frac{l^2}{E^2}=\frac{r_{\textrm{trap}}^2}{\xi (r_{\textrm{trap}})}$ by (2.18). As a result, we obtain

2.22 $$\begin{aligned} \frac{l^2}{E^2}-\frac{r_{\textrm{trap}}^2}{\xi (r_{\textrm{trap}})}=\frac{r^2}{\xi }\bigg (1-\frac{\xi }{r^2}\frac{r_{\textrm{trap}}^2}{\xi (r_{\textrm{trap}})}\bigg )-\frac{r^2}{\xi }\bigg (\frac{p^r}{E}\bigg )^2 \end{aligned}$$

due to (2.18). Then, if a geodesic is future or past trapped the normalised radial momentum $\frac{p^r}{E}$ satisfies

2.23 $$\begin{aligned} \frac{p^r}{E} = \pm \left( 1-\frac{\xi }{r^2}\frac{r_{\textrm{trap}}^2}{\xi (r_{\textrm{trap}})} \right) ^\frac{1}{2} =: \pm f(r). \end{aligned}$$

#### Ansatz for the multiplier

The function *f* defined by (2.23) is the *f* to be used in the current (2.11). Specifically, we obtain

2.24 $$\begin{aligned} f(r)&=\bigg (1-\dfrac{3M}{r}\bigg )\bigg (1+\dfrac{6M}{r}\bigg )^{\frac{1}{2}}, \quad \text {for Schwarzschild and }{n=4,} \end{aligned}$$

2.25 $$\begin{aligned} f(r)&=\bigg (1-\frac{2 \sqrt{M}}{r}\bigg ) \bigg (1+\frac{2 \sqrt{M}}{r}\bigg ), \quad \text {for Schwarzschild and } n=5, \end{aligned}$$

2.26 $$\begin{aligned} f(r)&= \bigg (1-\frac{2 M}{r}\bigg )\bigg (\frac{ r^2+4Mr -4 M^2}{ r^2}\bigg )^{\frac{1}{2}}, \quad \text {for extremal Reissner--Nordstr}\ddot{\text {o}}\text {m and }n=4, \end{aligned}$$

2.27 $$\begin{aligned} f (r)&= \frac{1}{\sqrt{1-27\frac{M^2}{L^2}}} \bigg (1-\frac{3M}{r}\bigg )\bigg (1+\frac{6M}{r}\bigg )^{\frac{1}{2}} ,\quad \text {for Schwarzschild--de Sitter and }n=4. \end{aligned}$$

We abstain from introducing a further subscript to distinguish the different *f*’s as it will always be clear from the context which *f* is to be used.

We close this section by collecting an important monotonicity property: There exists a constant $c>0$ depending only on the black hole parameters such that

2.28 $$\begin{aligned} f^\prime \ge c \frac{\xi }{r^3} \ \ \ \text {for (2.24), (2.25), (2.27),} \qquad \qquad f^\prime \ge c \left( 1-\frac{M}{r}\right) \frac{\xi }{r^3} \ \ \ \text {for (2.26).} \end{aligned}$$

## The main results

We recall the setting of Sect. 2.3 and the energy $\mathbb {E}^T[\psi ](\tau )$ defined in (2.14). We will state our Theorems for smooth solutions but by the usual density arguments the estimates also hold for appropriately defined weak solutions. When stating the results below we will use $A \lesssim B$ to mean $A \le C B$ for a constant *C* depending only on the black hole parameters.

### Theorem 1

(Integrated decay estimate on Schwarzschild [12]) Let $\phi$ be a solution of the wave equation $\Box _g \phi = 0$ for $(\mathcal {M},g)$ the exterior of a Schwarzschild black hole $\textrm{Schw}^{1+3}$. Then $\psi = \phi r$ satisfies the estimate3.1

Note that (3.1) is equivalent to the estimate replacing $\psi$ by $\phi$ and $\frac{dvol_{\mathcal {M}}}{r^{2}}$ by $dvol_{\mathcal {M}}$. We state the estimate for $\psi$ simply because it is easiest to prove in this form. While the estimate (3.1) has already been established in [12], we emphasise that our proof relies solely on the simple multiplier (2.24), which is independent of the spherical harmonic number. The estimates of [10, 28] are obtained with multipliers independent of the spherical harmonic number but—in comparison with (3.1)—either require higher order energies [10] or the current arising from a globally uniformly timelike vector field (instead of the Killing field $\partial _t$) on the right ([28], see also [34]).

Theorem 1 and its proof immediately generalise to the case of higher dimensional Schwarzschild metrics. We focus here on the $4+1$ dimensional case:

### Theorem 2

(Integrated decay estimate on 1+4 Schwarzschild) Let $\phi$ be a solution to the wave equation $\Box _g \phi =0$ for $(\mathcal {M},g)$ the exterior of a $1+4$ dimensional Schwarzschild black hole $\textrm{Schw}^{1+4}$. Then, $\psi =\phi r^\frac{3}{2}$ satisfies the estimate3.2

The estimate (3.2) in its exact form seems to be new. The paper [27] obtains (3.2) with the current arising from a uniformly timelike vector field on the right, while [32] requires higher order energies. See already Remark 6.1 for the higher dimensional case.

Our next result addresses solutions of (2.2) on the extremal Reissner–Nordström geometry [5, 21].

### Theorem 3

(Integrated decay estimate on extremal Reissner–Nordström) Let $\phi$ be a solution to the wave equation $\Box _g \phi =0$ the exterior of an extremal Reissner–Nordström black hole $\textrm{ERN}^{1+3}$. Then, $\psi = \phi r$ satisfies the estimate3.3

We note that the radial weights near infinity appearing in our main integrated estimates (3.1)–(3.3) are non-optimal even if one insists on only using the *T*-energy $\mathbb {E}[\psi ](0)$ on the right hand side. It is well-known how to optimise them with a multiplier localised to infinity. As mentioned in the introduction, if one is willing to use the redshift and weighted estimates near infinity, stronger estimates can be proven.

Our multiplier construction also produces an integrated local energy decay estimate for the conformal wave equation on the Schwarzschild–de Sitter exterior. In this case, there are two parameters involved and the resulting algebra is more involved. As a result, we only control the solution minus its spherical means. Note also that in this case, *r*-weights do not play any role as the exterior is compact in *r*.

### Theorem 4

(Integrated decay estimate on Schwarzschild–de Sitter) Let $\phi$ be a solution to the conformal wave equation $\Box \phi =\frac{2}{L^2}\phi$ on the exterior of a subextremal Schwarzschild–de Sitter black hole $\mathrm {SchwdS^{1+3}}$ which has vanishing spherical mean $\frac{1}{vol(\mathbb {S}^{2})}\int d\sigma _{\mathbb {S}^{2}} \psi \equiv 0$. Then, $\psi = \phi r$ satisfies the estimate3.4

The estimate of Theorem 4 seems to be new. See however [11], where a Morawetz estimate is proven for the *massless* wave equation on Schwarzschild–de Sitter using multipliers depending on the spherical harmonic number $\ell$.

### Remark 3.1

Since our integrated decay estimates in Theorems 1–4 only require the *T*-energy on the right hand side, they may find use in scattering theory applications. We refer to [15, 31] and references therein for a discussion of the fundamental role of the *T*-energy in black hole scattering theory and blueshift instabilities. The estimates in Theorem 1–4 may provide a useful tool to absorb errors when developing a scattering theory (based on the *T*-energy) on these backgrounds for non-linear wave equations.

## The proofs

We first note that it is sufficient to prove the estimates (3.1)–(3.4) without the $(\partial _t \psi )^2$-term on the left, which we will call the reduced estimate. Indeed, once the reduced estimate has been proven, a standard Lagrangian estimate recovers the missing derivative in terms of what has already been proven.

To prove the reduced estimate, we will integrate over $\mathcal {M}(\tau _1,\tau _2)$ the divergence identity (2.8) associated with the the current $C_f \cdot J^{(1)} + J^{(2)}$ with *f* being the *f* associated with the spacetime geometry under consideration and $C_f$ a constant to be fixed in Proposition 4.1 below. We define

4.1 $$\begin{aligned} \mathbb {E}^{aux}[\psi ](\tau )&:= 2\int _{\Sigma _\tau } \left( C_f \cdot J_\mu ^{(1)} + J_\mu ^{(2)}\right) n^\mu _{\Sigma _{\tau }} \, , \end{aligned}$$

4.2 $$\begin{aligned} \mathbb {I} \left[ \psi , f \right] (\tau _1,\tau _2)&:= 2 \int _{\mathcal {M}(\tau _1,\tau _2)} K^{(2)} dvol_{\mathcal {M}} \nonumber \\&= \int _{\mathcal {M}(\tau _1,\tau _2)} dt dr^\star d\sigma _{\mathbb {S}^{n-2}} \left[ 2f^\prime |\psi ^{\prime }|^2 - f \left( \frac{\xi }{r^2}\right) ^\prime |\nabla _{\mathbb {S}^{n-2}} \psi |^2 \right. \nonumber \\&\quad \left. -\left( f (V+\mu \xi )^\prime +\frac{f^{\prime \prime \prime }}{2}\right) |\psi |^2\right] . \end{aligned}$$

We have the following statement:

### Proposition 4.1

Let $\phi$ be a solution of $\Box _g \phi = 0$ for $(\mathcal {M},g)$ one of the spacetimes (1)–(3) above or a solution of $\Box _g \phi - \frac{2}{L^2}\phi = 0$ for the spacetime (4). Let the radial function *f* in the current $J^{(2)}$ satisfy

4.3 $$\begin{aligned} |f| + \bigg |\frac{r^2 f^\prime }{\xi }\bigg | + \bigg |\frac{r^3}{\xi } f^{\prime \prime }\bigg | \le C \, , \end{aligned}$$

for some *C* depending only on the black hole parameters. Then for any $\tau _2,\tau _1 \ge 0$

4.4 $$\begin{aligned} \mathbb {I} \left[ \psi , f \right] (\tau _1,\tau _2) \lesssim \mathbb {E}^T[\psi ](\tau _1) \, . \end{aligned}$$

### Proof

We recall (2.4) holds in all four cases considered. We claim that we can choose a $C_f$ depending only on parameters and the *C* in (4.3) such that both

4.5 $$\begin{aligned} \begin{aligned} c_1 J^{(1)}_\mu n_{\Sigma _\tau }^\mu&\le C_f J^{(1)}_\mu n_{\Sigma _\tau }^\mu + J^{(2)}_\mu n_{\Sigma _\tau }^\mu \phantom {xx} \le C_2 J^{(1)}_\mu n_{\Sigma _\tau }^\mu \, , \\ c_1 J^{(1)}_\mu (\partial _t)^\mu&\le C_f J^{(1)}_\mu (\partial _t)^\mu + J^{(2)}_\mu (\partial _t)^\mu \le C_2 J^{(1)}_\mu (\partial _t)^\mu \, , \end{aligned} \end{aligned}$$

hold for constants $c_1, C_2$ depending only on the parameters. This follows easily from the form of the currents (2.10) and (2.12) using (4.3), the bound (2.4) and the properties of $n_{\Sigma _\tau }$ defined in Sect. 2.2. We next apply the divergence identity (2.8) for the current $C_f \cdot J^{(1)} + J^{(2)}$ over $\mathcal {M}\left( \tau _1,\tau _2\right)$. It follows that

$$ \mathbb {I} \left[ \psi , f \right] (\tau _1,\tau _2) \le C_f \mathbb {E}^{aux}[\psi ](\tau _1) $$

as all boundary terms in the divergence identity except the one on $\Sigma _{\tau _1}$ have favourable signs. The right hand side is now immediately converted to $\mathbb {E}^{T}[\psi ](\tau _1)$ using the bounds (4.5). $\square$

Note that all *f*’s in (2.24)–(2.27) satisfy the assumption (4.3) hence (4.4) indeed holds. We next recall the form of (4.2), which is the left hand side of (4.4).

Denoting the spherical average of $\psi$ by $\psi _0:=\frac{1}{vol(\mathbb {S}^{n-2})}\int dvol_{\mathbb {S}^{n-2}} \psi$ and letting $g: [\rho _1,\rho _2) \rightarrow \mathbb {R}$ be a smooth function (with $g \cdot r$ uniformly bounded) to be chosen, we can write (4.2) as

$$\begin{aligned} \begin{aligned}&\mathbb {I} \left[ \psi , f \right] (\tau _1,\tau _2) \\&\quad =\int _{\mathcal {M}(\tau _1,\tau _2)} dt dr^\star d\sigma _{\mathbb {S}^{n-2}} \Bigg ( 2f^\prime |(\psi -\psi _0)^{\prime }|^2 - f \left( \frac{\xi (r)}{r^2}\right) ^\prime |\nabla _{\mathbb {S}^{n-2}} (\psi -{\psi }_0)|^2 \\&\qquad -\left( f (V+\mu \xi )^\prime +\frac{f^{\prime \prime \prime }}{2}\right) |\psi -{\psi }_0|^2 \Bigg ) +\int _{\mathcal {M}(\tau _1,\tau _2)} dt dr^\star d\sigma _{\mathbb {S}^{n-2}} \nonumber \\&\qquad \times \Bigg ( 2f^\prime |(\psi _0)^{\prime }+g\psi _0 |^2-\left( 2f^\prime g^2 -2(f^\prime g)^\prime + f (V+\mu \xi )^\prime +\frac{f^{\prime \prime \prime }}{2}\right) |{\psi }_0|^2 \Bigg ) \, . \end{aligned} \end{aligned}$$

Recall now the positivity property (2.28) and observe that all *f* in (2.24)–(2.27) satisfy $-f(r)\left( \frac{\xi (r)}{r^2}\right) ^\prime \ge 0$. (Specifically, recall that *f*(*r*) and $\left( \frac{\xi (r)}{r^2}\right) ^\prime$ both change sign at trapping.) Therefore, denoting by $\lambda ^{min}_{\mathbb {S}^{n-2}}$ the smallest non-trivial eigenvalue of the (negative) Laplacian on the unit-sphere, $-\Delta _{\mathbb {S}^{n-2}}$, it becomes apparent that the reduced estimates associated with (3.1), (3.2) and (3.4) will follow if *f* obeys the following two conditions:

4.6 $$\begin{aligned} - \lambda ^{min}_{\mathbb {S}^{n-2}} f \left( \frac{\xi (r)}{r^2}\right) ^\prime -\left( f (V+\mu \xi )^\prime +\frac{f^{\prime \prime \prime }}{2}\right) \ge c\frac{\xi }{r^3} \, , \end{aligned}$$

holds for a constant *c* depending only on parameters and given such an *f*, one can find a *g* such that also

4.7 $$\begin{aligned} -\left( 2f^\prime g^2 -2(f^\prime g)^\prime + f (V+\mu \xi )^\prime +\frac{f^{\prime \prime \prime }}{2}\right) \ge c\frac{\xi }{r^4} \end{aligned}$$

holds. Moreover, the reduced estimate associated with (3.3) will follow if (4.6) and (4.7) hold with $\xi$ replaced by $\left( 1-\frac{M}{r}\right) \xi$ on both right hand sides.

### Remark 4.1

Note that for solutions with vanishing spherical average, $\psi _0=0$, only (4.6) has to hold. This will be the case in the proof of Theorem 4.

### Remark 4.2

In the proof of Theorem 3 we will establish (4.7) with $\xi ^2$ instead of $\xi \left( 1-\frac{M}{r}\right)$, which leads to a slightly weaker estimate than (3.3), which is however easily improved a posteriori.

We complete the proof of the reduced estimate for (3.1)–(3.4) by checking (4.6) and (4.7) in each case.

### Proof of Theorem 1

We verify (4.6) with

$$\begin{aligned} n=4,\quad \xi =1-\frac{2M}{r},\quad \mu =0,\quad \lambda ^{min}_{\mathbb {S}^2} = 2 \end{aligned}$$

and

4.8 $$\begin{aligned} f(r)= \left( 1-\frac{3M}{r}\right) \sqrt{1+\frac{6M}{r}}, \quad \frac{d f}{dr} = \frac{27 M^2}{r^3 \sqrt{1+\frac{6 M}{r}}}>0. \end{aligned}$$

We compute

4.9 $$\begin{aligned}&- \lambda ^{min}_{\mathbb {S}^{2}} f \left( \frac{\xi (r)}{r^2}\right) ^\prime -\left( f V^\prime +\frac{f^{\prime \prime \prime }}{2}\right) =2\frac{2 \sqrt{\frac{6 M}{r}+1} (r-3 M)^2 (r-2 M)}{r^6} \nonumber \\&\qquad + \frac{M (r-2 M) \left( -54108 M^5+20628 M^4 r+5481 M^3 r^2-1182 M^2 r^3-176 M r^4+12 r^5\right) }{2 r^8 \sqrt{\frac{6 M}{r}+1} (6 M+r)^2} \nonumber \\&\quad = \frac{(r-2 M) \left( -54108 M^6+36180 M^5 r+2889 M^4 r^2-3342 M^3 r^3-104 M^2 r^4+108 M r^5+8 r^6\right) }{2 r^8 \sqrt{\frac{6 M}{r}+1} (6 M+r)^2}. \end{aligned}$$

It is easy to check (see the Appendix) that for $r\ge 2M$ we have

4.10 $$\begin{aligned} -54108 M^6+36180 M^5 r+2889 M^4 r^2-3342 M^3 r^3-104 M^2 r^4+108 M r^5+8 r^6 \ge c r^6 \end{aligned}$$

for some $c>0$ and hence that (4.6) holds.

To verify (4.7) we choose $g(r)=-\frac{1}{2} \left( 1-\frac{2M}{r}\right) \frac{1}{r} + \frac{1}{2} \frac{M^2}{r^3}$ and compute

4.11 $$\begin{aligned}&2\left( (f^\prime g)^\prime \!- f^\prime g^2 \right) -f V^\prime - \frac{1}{2} f^{\prime \prime \prime } \nonumber \\&\quad = \frac{M \sqrt{\frac{6 M}{r}+1} (r-2 M) \big (-972 M^7\!+\!21060 M^6 r\!-\!16551 M^5 r^2 \!+\!702 M^4 r^3\!+\!1215 M^3 r^4\!-\!48 M^2 r^5\!+\!13 M r^6\!+\!12 r^7\big )}{2 r^9 (6 M+r)^3}. \end{aligned}$$

It is now straightforward to conclude (see the Appendix) that for $r\ge 2M$ we have

4.12 $$\begin{aligned} -972 M^7+21060 M^6 r&-16551 M^5 r^2+702 M^4 r^3+1215 M^3 r^4\nonumber \\&-48 M^2 r^5+13 M r^6+12 r^7 \ge c r^7 \end{aligned}$$

for some constant $c>0$. Therefore, by simple asymptotic analysis we conclude that (4.7) holds.

### Proof of Theorem 2

We verify (4.6) with

$$\begin{aligned} n=5,\qquad \xi =1-\frac{2M}{r^2},\quad \mu =0,\quad \lambda ^{min}_{\mathbb {S}^3} = 3, \end{aligned}$$

and

4.13 $$\begin{aligned} f(r)= \frac{1}{r^2}\left( r-2 \sqrt{M}\right) \left( 2 \sqrt{M}+r\right) ,\quad \frac{df}{dr}= \frac{2 \sqrt{2}}{r^3}>0. \end{aligned}$$

We compute

4.14 $$\begin{aligned}&- \lambda ^{min}_{\mathbb {S}^{3}} f \left( \frac{\xi (r)}{r^2}\right) ^\prime -\left( f V^\prime +\frac{f^{\prime \prime \prime }}{2}\right) \nonumber \\&\quad = \frac{\frac{1}{ r^2} \left( r^2-2M\right) \left( -848 M^3 + 756 M^2 r^2 - 180 M r^4 + 15 r^6\right) }{4 \sqrt{2} r^9}. \end{aligned}$$

It is easy to check (see the Appendix) that for $r>\sqrt{2M}$ we have

4.15 $$\begin{aligned} -848 M^3 + 756 M^2 r^2 - 180 M r^4 + 15 r^6 \ge c r^6 \end{aligned}$$

for some $c>0$ which allows us to conclude (4.6).

To verify (4.7) we choose $g(r)=\frac{1}{2 r^3}-\frac{1-\frac{2 M}{r^2}}{2 r}$ and compute

4.16 $$\begin{aligned}&2\left( (f^\prime g)^\prime - f^\prime g^2 \right) -fV^\prime -\frac{1}{2}f^{\prime \prime \prime } \nonumber \\&\quad = \frac{\sqrt{\frac{1}{M r^4}} \left( r^2-2M\right) \left( -152 M^3+132 M^2 r^2-28 M r^4+3 r^6\right) }{4 \sqrt{2} r^9}. \end{aligned}$$

It is now straightforward to conclude (see the Appendix) that for $r \ge \sqrt{2M}$ we have

4.17 $$\begin{aligned} -152 M^3+132 M^2 r^2-28 M r^4+3 r^6 \ge c r^6 \, . \end{aligned}$$

for some constant $c>0$. Therefore, by simple asymptotic analysis we conclude that (4.7) holds.

### Proof of Theorem 3

We verify (4.6) with

4.18 $$\begin{aligned} n=4,\quad \xi =\left( 1-\frac{M}{r}\right) ^2,\quad \mu =0 ,\quad \lambda ^{min}_{\mathbb {S}^2} = 2, \end{aligned}$$

and

4.19 $$\begin{aligned} f(r)=\frac{(r-2 M) \sqrt{r (4 M+r)-4 M^2}}{r^2}, \quad \frac{d f}{dr} = \frac{(4 M)^2 (r-M)}{r^3 \sqrt{-4 M^2+4 M r+r^2}}\ge 0 . \end{aligned}$$

We compute

$$\begin{aligned} \begin{aligned}&- \lambda ^{min}_{\mathbb {S}^{2}} f \left( \frac{\xi (r)}{r^2}\right) ^\prime -\left( f V^\prime +\frac{f^{\prime \prime \prime }}{2}\right) \\&\quad = \frac{2M^6(r-M)^3}{M^3 r^{11} \sqrt{-4 M^2+2 r (2 M+r)-r^2} \left( 8 M^4+2 M^2 r^2-4 M^2 r (2 M+r)\right) ^2} \\&\qquad \times \Bigg (-1792 M^{10}+8960 M^9 r-17920 M^8 r^2+17920 M^7 r^3-8600 M^6 r^4\\&\qquad +712 M^5 r^5 +1056 M^4 r^6-312 M^3 r^7-43 M^2 r^8+19 M r^9+2 r^{10}\Bigg ). \end{aligned} \end{aligned}$$

It is easy to check (see the Appendix) that the polynomial

4.20 $$\begin{aligned} \begin{aligned}&-1792 M^{10}+8960 M^9 r-17920 M^8 r^2+17920 M^7 r^3-8600 M^6 r^4+712 M^5 r^5\\&\quad +1056 M^4 r^6-312 M^3 r^7-43 M^2 r^8+19 M r^9+2 r^{10} \end{aligned} \end{aligned}$$

is strictly positive for $r\ge M$. It then follows that $(4.20) \ge c \cdot r^{10}$ in $r \ge M$ for some $c>0$. (Indeed, fix $R>M$ large such that this holds in $r\ge R$ for any $c \le 1$. It then also holds in [*M*, *R*] for suitably small $c>0$ by compactness and the strict positivity already established.) We have concluded (4.6).

To verify (4.7), we choose $g(r)= -\frac{1}{2r}\left( 1-\frac{M}{r}\right) ^2+\frac{1}{2}\left( 1-\frac{M}{r}\right) \frac{M^2}{r^3}$ and compute

4.21 $$\begin{aligned} 2\left( (f^\prime g)^\prime - f^\prime g^2 \right) -fV^\prime -\frac{1}{2}f^{\prime \prime \prime } = \frac{(r-M)^4}{2 r^{13} \left( -4 M^2+4 M r+r^2\right) ^{5/2}}p(r) \, , \end{aligned}$$

where

4.22 $$\begin{aligned} \begin{aligned} p(r)&=64 M^{10}-1216 M^9 r+4640 M^8 r^2-7200 M^7 r^3+4788 M^6 r^4\\&\quad -732 M^5 r^5-444 M^4 r^6+92 M^3 r^7+4 M^2 r^8+4 M r^9+3 r^{10}. \end{aligned} \end{aligned}$$

It is now straightforward (see the Appendix) to conclude that for some $c>0$ the polynomial *p*(*r*) satisfies

4.23 $$\begin{aligned} p(r) \ge c \cdot r^{10},\quad r \ge M. \end{aligned}$$

We concluded that the estimate (4.7) holds with $\xi ^2$ instead of $\xi \left( 1-\frac{M}{r}\right)$ on the right hand side and leads to (3.3) except that the zeroth order term has an additional $(1-\frac{M}{r})$-degeneration at the horizon. Another Hardy type inequality using the good $(\partial _{r^\star } \psi )^2$-term easily removes this additional degeneration a posteriori and the proof is complete.

### Proof of Theorem 4

Note that in the present proof we only verify (4.6), but not condition (4.7), see the relevant Remark 4.1.

We verify (4.6) with

$$\begin{aligned} n=4,\quad \xi =1-\frac{2M}{r}-\frac{r^2}{L^2},\quad \mu =\frac{2}{L^2},\quad \lambda ^{min}_{\mathbb {S}^2} = 2 , \end{aligned}$$

where $$L^2=\frac{3}{\Lambda }$$ and

$$\begin{aligned} f(r)= \frac{1}{\sqrt{1-27\frac{M^2}{L^2}}}\left( 1-\frac{3M}{r}\right) \sqrt{1+\frac{6M}{r}} , \quad \frac{d f}{dr} = \frac{1}{\sqrt{1-27\frac{M^2}{L^2}}}\frac{27 M^2}{r^3 \sqrt{1+\frac{6 M}{r}}}>0. \end{aligned}$$

We compute

$$\begin{aligned} - \lambda ^{min}_{\mathbb {S}^{2}} f \left( \frac{\xi (r)}{r^2}\right) ^\prime -\left( f (V+\mu \xi )^\prime +\frac{f^{\prime \prime \prime }}{2}\right) = \frac{1}{2r^7(r+6M)^2\sqrt{1-27\frac{M^2}{L^2}}\sqrt{1+\frac{6M}{r}}} \xi S(r), \end{aligned}$$

where

4.24 $$\begin{aligned} \begin{aligned} S(r)&= -\frac{4 M \left( 4212 M^4+702 M^3 r-189 M^2 r^2-39 M r^3+r^4\right) r^3}{L^2} +\frac{81 M^3 (3 M+2 r) r^6}{L^4}\\&\quad -54108 M^6+28404 M^5 r+4185 M^4 r^2-2262 M^3 r^3-140 M^2 r^4+60 M r^5+4 r^6. \end{aligned} \end{aligned}$$

We note that

$$\begin{aligned} 0<\frac{M^2}{L^2}<\frac{1}{27},\qquad 2M<r_+<r<L , \end{aligned}$$

where the last inequality is immediate from that $\xi (2M)<0$, $\xi (L)<0$.

Moreover, the polynomial

$$\begin{aligned} -54108 M^6+28404 M^5 r+4185 M^4 r^2-2262 M^3 r^3-140 M^2 r^4+60 M r^5+4 r^6 \end{aligned}$$

is strictly increasing, and specifically

$$\begin{aligned} -54108 M^6+28404 M^5 r+4185 M^4 r^2-2262 M^3 r^3-140 M^2 r^4+60 M r^5+4 r^6\ge 1280M^6, \end{aligned}$$

where $1280 M^6$ is its value at $r=2M$. For simplicity, we denote

$$\begin{aligned} 1-\frac{2M}{r}-\frac{r^2}{L^2}=\xi (r) >0 \quad \implies \quad r^3 = L^2 (r-2M-\xi (r) r) \, . \end{aligned}$$

We rewrite *S*(*r*) as follows

4.25 $$\begin{aligned} \begin{aligned} S(r)&= \xi ^2(r) \left( 243 M^4 r^2+162 M^3 r^3\right) + 4 (3 M-r)^2 (6 M+r)^2 \left( r^2-15 M^2+8 M r\right) \\&\quad +\xi (r) \left( 17820 M^5 r+2970 M^4 r^2-1080 M^3 r^3-156 M^2 r^4+4 M r^5\right) , \end{aligned} \end{aligned}$$

where we note that $r^2+8Mr-15M^2>0$ for $r\in [r_+,\bar{r}_+]$.

Now, suppose that there exists $r_0\in [r_+,\bar{r}_+]$ such that

4.26 $$\begin{aligned} \left( 17820 M^5 r+2970 M^4 r_0^2-1080 M^3 r_0^3-156 M^2 r_0^4+4 M r_0^5\right) <0, \end{aligned}$$

otherwise $S(r)>0$. Then, by using that $\xi (r_0)<1$ we bound $S(r_0)$ from below

$$\begin{aligned} \begin{aligned} S(r_0)&> \left( 17820 M^5 r+2970 M^4 r_0^2-1080 M^3 r_0^3-156 M^2 r_0^4+4 M r_0^5\right) \\&\quad + 4 (3 M-r_0)^2 (6 M+r_0)^2 \left( r_0^2-15 M^2+8 M r_0\right) = -19440 M^6\\&\quad +34668 M^5 r_0 +2430 M^4 r_0^2-2736 M^3 r_0^3-132 M^2 r_0^4+60 M r_0^5+4 r_0^6. \end{aligned} \end{aligned}$$

A direct critical point analysis (see the Appendix) of the polynomial

4.27 $$\begin{aligned} \tilde{p}(r)=-19440 M^6+34668 M^5 r+2430 M^4 r^2-2736 M^3 r^3-132 M^2 r^4+60 M r^5+4 r^6 \end{aligned}$$

establishes that for $r>2M$, we have $\tilde{p}(r)> 0$, which proves that even for $r_0$ values such that (4.26) holds we obtain that $S(r_0)>0$. We have therefore concluded (4.6) and the proof.

## Normal hyperbolicity and commutator vector fields

In this section, we elucidate the connection between the multipliers of the present paper and the globally good commutators introduced in [25, 29]. For this, we continue our discussion about trapped null geodesics from Sect. 2.6, but restricting now to a Schwarzschild spacetime. This is merely for simplicity of the expressions. Identical considerations apply—with trivial algebraic changes—to the spacetimes (1)–(4). Our goal is to show that the globally good commutators introduced in [25, 29] arise by studying the expansion and contraction properties of the geodesic flow.

### Stable manifolds and commutator vector fields

Let us discuss the hyperbolic properties and stable manifolds associated to the trapped set $\Gamma$ defined in (2.21).

We define the *stable *($W^+$) *and unstable* ($W^-$) *sets associated to the trapped set* $\Gamma$ as

$$\begin{aligned} W^+&:=\big \{(x,p)\in \mathcal {P}: (r(s),p^r(s))\rightarrow (r_{\textrm{trap}},0)\quad \text {as} \quad s\rightarrow +\infty \big \}, \\ W^-&:=\big \{(x,p)\in \mathcal {P}: (r(-s),p^r(-s))\rightarrow (r_{\textrm{trap}},0)\quad \text {as} \quad s\rightarrow +\infty \big \}. \end{aligned}$$

By the stable manifold theorem for normally hyperbolic sets [23], a generalisation of the Hadamard–Perron theorem, the stable and unstable sets are, locally around $\Gamma$, codimension one submanifolds of $\mathcal {P}$. See [26, Section 6.2] for a modern reference. These are the *stable manifolds associated to* $\Gamma$. For Schwarzschild (and in fact any of the spacetimes (1)–(4)), we can give a self-contained proof that $W^{\pm }$ are *global* codimension one submanifolds of $\mathcal {P}$:

#### Proposition 5.1

(Characterisation of the stable sets) The stable and unstable sets associated to the trapped set in the exterior of Schwarzschild spacetime are characterised as

5.1 $$\begin{aligned} W^{\pm }=\bigg \{(x,p)\in \mathcal {P}:\dfrac{l}{E}=3\sqrt{3}M,~ \frac{p^{r}}{E}=\pm \bigg (1+\dfrac{6M}{r}\bigg )^{\frac{1}{2}}\bigg (1-\dfrac{3M}{r}\bigg ) \bigg \}. \end{aligned}$$

In particular, $W^{+}$ and $W^{-}$ are smooth codimension one submanifolds of $\mathcal {P}$ such that $W^+\cap W^-=\Gamma$.

We first prove some lemmata that will be used in the proof of this proposition. We begin showing that for any $(x,p)\in \mathcal {P}$ with $\frac{l^2}{E^2}- 27\,M^2\ne 0$, $(r(s),p^r(s))$ does not converge to $(r_{\textrm{trap}},0)$ as $s\rightarrow \pm \infty$. We note by (2.18) that the radial momentum $p^r$ satisfies

5.2 $$\begin{aligned} (p^r)^2=\frac{E^2}{r^3}\bigg (r^3-\frac{l^2}{E^2}(r-2M)\bigg ). \end{aligned}$$

We start considering the case when $\frac{l^2}{E^2}- 27\,M^2>0$. Note by (5.2) that along any geodesic $\gamma$, we have

5.3 $$\begin{aligned} \frac{l^2}{E^2}\le \frac{r^3(s)}{r(s)-2M},\qquad \forall s\in \mathbb {R}. \end{aligned}$$

#### Lemma 5.1

Let $\epsilon >0$. Every geodesic $\gamma$ with $\frac{l^2}{E^2}> 27\,M^2$ is either contained in $\{r<3\,M\}$ or $\{r>3\,M\}$. Moreover, if $\gamma$ is contained in $\{r<3\,M\}$ with $\frac{l^2}{E^2}> 27\,M^2+\epsilon$, then $r(s)\le (2+\frac{27\,M^2}{27\,M^2+\epsilon })M$ for all $s\in \mathbb {R}.$ Otherwise, if $\gamma$ is contained in $\{r>3\,M\}$ with $\frac{l^2}{E^2}\ge 27\,M^2+\epsilon$, then $r(s)\ge (27\,M^3+\epsilon M)^{\frac{1}{3}}$ for all $s\in \mathbb {R}.$

#### Proof

From (5.3), geodesics with $\frac{l^2}{E^2}> 27\,M^2$ cannot have $r(s)=3\,M$, so every such geodesic is either in $\{r<3\,M\}$ or $\{r>3\,M\}$. By (5.3), every geodesic in $\{r<3\,M\}$ with $\frac{l^2}{E^2}> 27\,M^2+\epsilon$ satisfies $r(s)\le \frac{E^2}{l^2}r^3+2\,M\le (2+\frac{27\,M^2}{27\,M^2+\epsilon })M.$ By (5.3) again, every geodesic in $\{r>3\,M\}$ with $\frac{l^2}{E^2}\ge 27\,M^2+\epsilon$ satisfies $r^3(s)\ge \frac{l^2}{E^2}(r-2\,M)\ge 27\,M^3+\epsilon M.$ $\square$

Let us now consider the cases when $(x,p)\in \mathcal {P}$ satisfies $\frac{l^2}{E^2}- 27\,M^2<0$ and $\frac{l^2}{E^2}- 27\,M^2=0$, separately.

#### Lemma 5.2

Let $0<\epsilon \le 27\,M^2$. For every geodesic $\gamma$ with $\frac{l^2}{E^2}< 27\,M^2$, we have $|p^r(s)|> 0$ for all $s\in \mathbb {R}$. Moreover, if $\gamma$ has $\frac{l^2}{E^2}\le 27\,M^2-\epsilon$, then $(p^r(s))^2\ge \epsilon E^2\frac{(r(s)-2\,M)}{r^3(s)}$ for all $s\in \mathbb {R}.$

#### Proof

By (5.2), the result follows by $(p^r)^2=E^2\frac{(r+6\,M)(r-3\,M)^2}{r^3}+\epsilon E^2\frac{(r-2\,M)}{r^3}\ge \epsilon E^2\frac{(r-2\,M)}{r^3}.$ $\square$

#### Lemma 5.3

Every null geodesic $\gamma$ with $\frac{l^2}{E^2}=27\,M^2$, is either contained in $\{r<3\,M\}$, $\{r=3\,M\}$, or $\{r>3\,M\}$. Moreover, for every such geodesic in $\{r<3\,M\}$ or $\{r>3\,M\}$, we have $|p^r(s)|> 0$ for all $s\in \mathbb {R}$, and additionally $(r(s),p^r(s))\rightarrow (3\,M,0)$ as $s\rightarrow +\infty$, or $(r(-s),p^r(-s))\rightarrow (3\,M,0)$ as $s\rightarrow +\infty$.

#### Proof

By the identity (5.2) and $\frac{l^2}{E^2}=27\,M^2$, we have

5.4 $$\begin{aligned} \bigg (\frac{dr}{ds}\bigg )^2=\frac{E^2}{r^3}(r+6M)(r-3M)^2. \end{aligned}$$

This ODE is satisfied by the constant solution $r(s)\equiv 3\,M$ for which $p^r\equiv 0$. And if one solves this ODE with an initial datum $r(s=0)=r_0$ such that $r_0\ne 3\,M$, necessarily the corresponding geodesic is either contained in $\{r<3\,M\}$ or $\{r>3\,M\}$. Note that by (5.4), we have $(p^r)^2> 0$ for all $r\in (2\,M,+\infty ){\setminus } \{3\,M\}$.

For the last part of the lemma, we use separation of variables to integrate the (5.4). Let us consider a null geodesic $\gamma$ in $\{r<3\,M\}$ with $\frac{l^2}{E^2}=27\,M^2$. We suppose that $\gamma$ is outgoing, meaning that $p^r(s)>0$ for all $s\in \mathbb {R}$. By integrating (5.2) with initial datum $r=r_0$ for some $r_0\in (2\,M,3\,M)$, we have

5.5 $$\begin{aligned} Es= \int _{r_0}^{r(s)}\frac{r^{\frac{3}{2}}dr}{(r+6M)^{\frac{1}{2}}(3M-r)}, \end{aligned}$$

where we have assumed that $r_0=r(s=0)$ for simplicity. We have used $\gamma (\mathbb {R})\subset \{r<3\,M\}$ in the sign of the integral above. By an explicit computation, one can integrate the RHS above as

$$\begin{aligned} \int \frac{r^{\frac{3}{2}}dr}{(r+6M)^{\frac{1}{2}}(3M-r)}&=2\sqrt{3}M\log \bigg (1+\sqrt{\frac{3r}{6M+r}}\bigg )\nonumber \\  &\quad +\sqrt{3}M\log \bigg (\frac{6M+r}{2(3M-r)}\bigg )-\sqrt{r(r+6M)}+C=:I(r), \end{aligned}$$

where $C\in \mathbb {R}$ is an arbitrary constant. As a result, we use (5.5) to obtain the estimate

$$\begin{aligned} Es&=I(r(s))-I(r_0)\le 2\sqrt{3}M\log \bigg (1+\sqrt{\frac{3r}{6M+r(s)}}\bigg )\\  &\quad +\sqrt{3}M\log \bigg (\frac{6M+r(s)}{2(3M-r(s))}\bigg )+C-I(r_0), \end{aligned}$$

where the 1st term in the RHS is bounded since $\gamma (\mathbb {R})\subset \{r<3\,M\}$. Then, $r(s)\rightarrow 3\,M$ as $s\rightarrow +\infty$. And we obtain $\lim _{s\rightarrow \pm \infty }p^r(s)= 0$ by using (5.4). One can similarly treat the case when $\gamma$ is ingoing instead, and also the cases when $\gamma$ is contained in $\{r>3\,M\}$. $\square$

We are now ready to prove Proposition 5.1.

#### Proof of Proposition 5.1

By Lemmata 5.1–5.2, for any $(x,p)\in \mathcal {P}$ with $\frac{l^2}{E^2}- 27\,M^2\ne 0$, $(r(s),p^r(s))$ does not converge to $(r_{\textrm{trap}},0)$ as $s\rightarrow \pm \infty$. Putting together Lemmata 5.1–5.3, for all $(x,p)\in W^+\cup \,W^{-}$ there holds $\frac{l^2}{E^2}=27\,M^2$. Using (5.2) with $\frac{l^2}{E^2}=27\,M^2$, and suitably choosing the sign of $p^r$, the result follows.

Finally, we observe that $\mathcal {P}$ is $7$-dimensional. The sets $W^\pm$ can be parametrised by an arbitrary $x\in \mathcal {M}$, and a momentum *p* which can in turn be parametrised by 2 parameters, namely *l* and $p^{\theta }$ such that $(p^{\theta })^2 + \sin ^2(\theta )(p^{\phi })^2=l^2/r^2$. Here, *E* is determined by $l^2/E^2=27M^2$ and $p^r$ by (5.4). So $\mathcal {P}$ is indeed 6-dimensional as claimed. The smoothness of $W^{\pm }$ follows by the smoothness of this parametrisation. $\square$

We finally write the sets $W^{\pm }$ in terms of suitable defining functions $\varphi _{\pm }$. Set the functions $\varphi _{\pm }\,:\mathcal {P}\rightarrow \mathbb {R}$,

$$\begin{aligned} \varphi _{\pm }(x,p):=\frac{r^\frac{3}{2}}{(r-2M)^{\frac{1}{2}}}\bigg (1+\frac{6M}{r}\bigg )^{\frac{1}{2}}\bigg (1-\frac{3M}{r}\bigg )\pm \frac{r^\frac{3}{2}}{(r-2M)^{\frac{1}{2}}}\bigg (\frac{p^r}{E}\bigg ). \end{aligned}$$

Note that $\frac{l^2}{E^2}-27M^2=\varphi _{+}\varphi _{-}$ by (2.22). In terms of the functions $\varphi _{\pm }$, the stable sets can be written as

$$\begin{aligned} W^+=\big \{ (x,p)\in \mathcal {P}:\varphi _{-}(x,p)=0\Big \},\qquad W^-=\big \{ (x,p)\in \mathcal {P}:\varphi _{+}(x,p)=0\big \}. \end{aligned}$$

The normal hyperbolicity of the trapped set $\Gamma$ is expressed, at the level of the geodesic flow, in the following proposition in terms of the defining functions $\varphi _{\pm }$.

#### Proposition 5.2

(Expansion and contraction of the geodesic flow) The derivative of $\varphi _{\pm }$ along the null geodesic flow in Schwarzschild spacetime is

5.6 $$\begin{aligned} \frac{d}{ds}\varphi _{\pm }=\pm \frac{1}{(1-\frac{2M}{r})r(1+\frac{6M}{r})^{\frac{1}{2}}}E\varphi _{\pm }. \end{aligned}$$

#### Proof

Follows by elementary calculations using the radial geodesic Eq. (2.19). $\square$

Let $\pi \,:\mathcal {P}\rightarrow \mathcal {M}$ be the canonical projection defined by $\pi (x,p)=x$. We consider suitably normalised normals $V_{\pm }$ of the 3-dimensional submanifolds $\pi (W^{\pm })\subset \mathcal {M}$. Given a momentum $p\in T_x\mathcal {M}$ at $x$, we see

5.7 $$\begin{aligned} V_\pm :=\dfrac{r^{\frac{3}{2}}}{(r-2M)^{\frac{1}{2}}}\bigg (\pm \bigg (1+\dfrac{6M}{r}\bigg )^{\frac{1}{2}}\bigg (\dfrac{3M}{r}-1\bigg )\,\partial _t+\partial _{r_*}\bigg )\quad \text {is such that} \,\, g(V_\pm ,p)=\varphi _\mp . \end{aligned}$$

By considering the vector fields $V_{\pm }$ in a suitable moving frame, one can show the expansion and contraction properties of the differential of the flow map $d\phi ^t_{(x,p)}$ by studying the Jacobi equation. This is related to the *normal hyperbolicity of the trapped set of Schwarzschild*. See Sect. 5.3 for more information. A detailed discussion of the differential of the geodesic flow is beyond the scope of this paper, see [18, 37].

### Commutation formula with the wave operator

The vectorfield $V_-$ above satisfies good commutation properties with the wave operator $\Box _{g}$ on Schwarzschild [25, 29]. The commutation of $V_-$ with $\Box _{g}$ corresponds, in the high frequency regime, to the Poisson bracket with $\varphi _{+}$ computed in Proposition 5.2. For comparison, we state the commutation formula in [25, equation (12)] and [29, Proposition 4.1].

#### Proposition 5.3

Let $\phi$ be a solution to the wave equation on $\textrm{Schw}^{1+3}$. Then,

5.8 $$\begin{aligned} \Box _g (V_-\phi )&=[\Box _g,V_- ]\phi =\frac{2}{(1-\frac{2M}{r})r(1+\frac{6M}{r})^{\frac{1}{2}}}\partial _tV_-\phi \nonumber \\  &\quad + E_1(r)\partial _t\phi + E_2(r)\frac{1}{1-\frac{2M}{r}}\left( \partial _{r^\star }+f(r)\partial _t\right) \phi , \end{aligned}$$

where

5.9 $$\begin{aligned} \begin{aligned} E_1(r)&=-\frac{2(1-\frac{2M}{r})^{\frac{1}{2}}}{r}, \quad E_2(r) = \frac{M^2}{r^3(1-\frac{2M}{r})^{\frac{1}{2}}},\\ f(r)&= \bigg (1-\frac{3M}{r}\bigg )\bigg (1+\frac{6M}{r}\bigg )^{\frac{1}{2}}. \end{aligned} \end{aligned}$$

Using Proposition 5.3, [25] obtains local integrated decay estimates for the perturbed wave equation $\Box _{g}\phi =\epsilon \beta ^{a}\partial _a \phi$ on Schwarzschild, where $\beta$ is a regular vectorfield suitably decaying in space. And [29] proves relatively non-degenerate integrated energy estimates for the wave equation on Schwarzschild–de Sitter using a more general commutation formula. Extensions of Proposition 5.3 have been used for the study of local integrated decay estimates on Kerr [24] and Kerr–de Sitter spacetimes [30].

### Normal hyperbolicity of the trapped set of black holes

As mentioned in the beginning of Sect. 5.1, in the language of hyperbolic dynamical systems, the existence and properties of the stable manifolds $W^{\pm }$ and the vector fields $V_{\pm }$ are a consequence of the $r$-normal hyperbolicity for all $r$ of the trapped set of Schwarzschild in the sense of [20, Section II]. We abstain from giving the general definition here (as it is not needed for the paper) and refer the reader [23] for a general discussion of this notion. We note however that the normal hyperbolicity property of the trapped set of Schwarzschild (and very slowly rotating Kerr spacetimes) was shown in [37], and later extended to Schwarzschild–de Sitter (and very slowly rotating Kerr–de Sitter) [36]. Similar arguments apply to $\textrm{ERN}^{1+3}$. The fact that normal hyperbolicity can be established for more general black hole backgrounds suggests that the construction of the vector fields $V^\pm$ (obtained by explicit calculation for Schwarzschild in Sect. 5.1) applies in much greater generality. By the stable manifold theorem for normally hyperbolic sets [23], the normal hyperbolicity of the trapped set $\Gamma$ implies that there are, locally around $\Gamma$, suitable stable manifolds $W^\pm$, which can be written (locally again) as the zero set of suitable functions $\varphi _\pm$. See also [26, Section 6.2]. A (generally pseudodifferential) version of the vector fields $V_\pm$ can then be defined as follows: One considers a normal to $W^\pm \subset \mathcal {P}$ and projects it (at each fixed $(x,p) \in W^\pm$) to $T_x \mathcal {M}$ producing a “vector field” $V_\pm$ which one normalises by demanding $g_x(V_\pm ,p)=\varphi _{\mp }$ for a given momentum $p\in T_x\mathcal {M}$. Note that the name vector field for the $V_\pm$ is indeed abusive as their coefficients may depend on the momentum variables and the operator is thus pseudodifferential. For an illustration see [24, 30], where a pseudodifferential generalisation of the vector fields (5.7) is constructed for subextremal Kerr and Kerr–de Sitter spacetimes.

## Conclusion and final remarks

The results of the paper provide both short proofs and a unifying perspective on integrated decay estimates for various different black hole backgrounds. However, they still rather provide a “recipe” that then needs to be checked for each individual spacetime by computation, rather than a conceptual proof that this recipe *always* leads to the desired estimate for a suitably defined class of spacetimes.

To nevertheless put the results in a more general context we note that the recipe itself can be applied for any static, spherically symmetric black hole exterior with non-trivial trapping. Indeed, if the radial momentum $p^r$ of a null geodesic that starts at distance *r* with energy (corresponding to the conserved quantity associated with the static vector field) $E=1$ and eventually becomes future trapped, defines a unique sufficiently smooth function on the black hole exterior, then we can set $f(r)=p^r(r)$ and apply the Morawetz current defined in Section 2 of the paper. The conjecture is that this choice will produce an integrated local energy decay estimate for the covariant wave equation on the chosen black hole exterior.

The following three remarks provide a few more examples:

### Remark 6.1

(*On higher dimensional Schwarzschild*) Let

$$\begin{aligned} g = -\bigg (1-\frac{2M}{r^{n-3}}\bigg )dt \otimes dt +\bigg (1-\frac{2M}{r^{n-3}}\bigg )^{-1}dr \otimes dr +r^2 d\sigma _{\mathbb {S}^{n-2}}, \end{aligned}$$

where $n\ge 4$, be the metric of an *n* dimensional Schwarzschild black hole. For more information about this specific geometry see [32]. Motivated by the considerations of Sect. 2.6, we define the multiplier

$$\begin{aligned} \begin{aligned} f(r)&=\frac{r- r_{\textrm{trap}}}{r^{\frac{n-1}{2}}}\\  &\quad \times \sqrt{ \sum _{k=2}^{n-1} \frac{-(r- r_{\textrm{trap}})^{k-2}}{k!} \partial _{r}^k\Big |_{r= r_{\textrm{trap}}}\left( -( r_{\textrm{trap}})^{-2} r^{n-1}+2 M \left( r_{\textrm{trap}}\right) ^{1-n} r^{n-1}-2 M+r^{n-3}\right) }, \end{aligned} \end{aligned}$$

where $r_{\textrm{trap}} =(n-1)^{\frac{1}{n-3}} M^{\frac{1}{n-3}}$ is the value that defines the corresponding photon sphere. We expect that by using this *f* in (4.4), one produces a coercive estimate also for $n\ge 6$.

### Remark 6.2

(*On subextremal Reissner–Nordström*) Let

$$\begin{aligned} g =-\left( 1-\frac{2M}{r}+\frac{Q^2}{r^2}\right) dt \otimes dt +\left( 1-\frac{2M}{r}+\frac{Q^2}{r^2}\right) ^{-1} dr \otimes dr + r^2 d\sigma _{\mathbb {S}^2} \end{aligned}$$

be the metric of a subextremal Reissner–Nordström black hole $|Q|<M$. Motivated by the considerations of Sect. 2.6, we define the multiplier

6.1 $$\begin{aligned} \begin{aligned} f(r) = \frac{1}{r}\bigg (1-\dfrac{ r_{\textrm{trap}}}{r}\bigg )\bigg (r^2+2 r_{\textrm{trap}} r-\dfrac{ r_{\textrm{trap}}^2Q^2}{\Delta ( r_{\textrm{trap}})}\bigg )^{\frac{1}{2}}, \quad \Delta (r)=r^2-2Mr+Q^2, \end{aligned} \end{aligned}$$

where

$$\begin{aligned} r_{\textrm{trap}}= \frac{1}{2} \left( \sqrt{9M^2-8Q^2}+3M\right) \end{aligned}$$

is the value that defines the corresponding photon sphere. The authors have verified for several subextremal parameters that the multiplier above produces coercive estimates for solutions with vanishing spherical mean. With more effort, one should be able to check the entire subextremal range.

### Remark 6.3

(*On subextremal Reissner–Nordström–de Sitter*) Let

$$\begin{aligned} g =-\left( 1-\frac{2M}{r}+\frac{Q^2}{r^2}-\frac{r^2}{L^2}\right) dt\otimes dt +\left( 1-\frac{2M}{r}+\frac{Q^2}{r^2}-\frac{r^2}{L^2}\right) ^{-1} dr\otimes dr + r^2 d\sigma _{\mathbb {S}^2} \end{aligned}$$

be the metric of a Reissner–Nordström–de Sitter black hole, where $L^2=\frac{3}{\Lambda }$ is defined in terms of the cosmological constant $\Lambda > 0$. To the best of our knowledge a Morawetz estimate for the wave equation on Reissner–Nordström–de Sitter $(\Lambda >0)$ has not been proved in the literature. It would be interesting to understand further the Morawetz estimate produced by the multiplier suggested from the considerations of Sect. 2.6, in a near extremal Reissner–Nordström–de Sitter black hole, where a violation of Strong Cosmic Censorship has been conjectured [9]. Specifically, it would be interesting to track down the constants in the Morawetz estimate and the relevant commutator that gives a ‘relatively non-degenerate estimate’. See [29] for the proof of a ‘relatively non-degenerate estimate’ on Schwarzschild–de Sitter.

Finally, natural generalisations of the method to non-spherically symmetric stationary spacetimes suggest itself. For instance, let us consider axisymmetric solutions of $\Box _g \phi =0$ on a (sub)extremal Kerr exterior. Restricting to axisymmetric null geodesics and determining the radial momentum of those which become future trapped (using the integrability of geodesic flow) produces a unique function *f*(*r*) and hence a candidate multiplier in this setting. Checking the positivity of the relevant expressions for all parameters (*M*, *a*) seems computationally very involved even though numerical experiments indicate that it indeed holds. More generally, restricting to null geodesics with fixed energy *E* (corresponding to the Killing field *T*) and azimuthal number *m* (corresponding to the Killing field $\Phi$) there is again a unique function *f*(*r*, *E*, *m*) corresponding to future trapped null geodesics. Using the correspondence of such geodesics with solutions $\phi$ of the wave equation with fixed time frequency $\omega \sim E$, the procedure now determines pseudodifferential multipliers with potentially good coercivity properties. This opens the possibility of proving integrated decay estimates without using the full Carter separation of the wave equation [14]. We leave a systematic investigation of this to future work.

## Data Availability

Data sharing is not applicable to this article as no datasets were generated nor considered in this research.

## Appendix A: Establishing positivity of polynomials

For the reader’s convenience, we provide simple calculus arguments establishing rigorously the positivity of all seven polynomials appearing in the proofs of the theorems.

### A.1 Theorem 1

For (4.10), after setting $r=2Mx$ it suffices to show ($c=\frac{1}{64}$) positivity of the polynomial

$$ p(x) = -54108 + 72360 x + 11556 x^2 - 26736 x^3 - 1664 x^4 + 3456 x^5 + 511 x^6 \ \ \ \text {in } x \ge 1, $$

where note that we rescaled with $M^6$. Clearly $p(1)=5376\ge 1$. We will show $p(x) \ge 1$ for all $x\ge 1$ by showing $p^\prime (x) \ge 0$ for all $x \ge 1$. Indeed,

$$\begin{aligned} p^\prime (x)&= 72360 + 23112 x - 80208 x^2 - 6656 x^3 + 17280 x^4 + 3066 x^5 \\&= 3066 x \left( x^2 - \sqrt{\frac{23112}{3066}}\right) ^2 + 17280 \left( x^2 - \sqrt{\frac{72360}{17280}}\right) ^2 + ax^3 + bx^2 \, , \end{aligned}$$

with $a=2\sqrt{3066 \cdot 23112} - 6656$ and $b= 2\sqrt{17280 \cdot 72360}-80208$. Since $a>|b|$ and $x^3 \ge x^2$ we have $p^\prime (x) \ge 0$.

For the second polynomial (4.12), after setting again $r=2Mx$, it suffices to show ($c=\frac{1}{32}$)

$$\begin{aligned} q(x)&= -243 + 10530 x - 16551 x^2 + 1404 x^3 + 4860 x^4 \\  &\quad - 384 x^5 + 208 x^6 + 383 x^7 \ge 0 \ \ \ \text {in } x \ge 1, \end{aligned}$$

where note that we rescaled with $M^7$. We easily compute $q(1)=207$, $q^\prime (1)=3089$ and establish positivity of $q^{\prime \prime }(x)$ for all $x \ge 1$:

$$\begin{aligned} q^{\prime \prime }(x)&=-33102 + 8424 x + 58320 x^2 - 7680 x^3 + 6240 x^4 \\  &\quad + 16086 x^5 \ge -33102+66744x \ge 0 \, \ \text {for all }x\ge 1. \end{aligned}$$

### A.2 Theorem 2

For (4.15), after setting $r=\sqrt{2M}x$, it suffices to observe ($c=\frac{1}{2}$) that

$$ p(x)= -212 +378 x^2 -180 x^4 +29 x^6\ge 0 \ \ \ \text {for }x\ge 1, $$

since $p(1)=15$ while $p^\prime (x)=756 x-720x^3 +174 x^5 = 174x \left( x^2-\sqrt{\frac{756}{174}}\right) ^2+(2\sqrt{756\cdot 174}-720)x \ge 0$ for $x \ge 1$.

For the second polynomial (4.17), after setting $r=\sqrt{2M}x$, it suffices to observe ($c=1$) that

$$ q(x)= -19 +33 x^2 -14 x^4 +2x^6 \ge 0 \ \ \ \text {for }x\ge 1, $$

since $q(1)=2$ and $q^\prime (x)=66x -56x^3 +12 x^5 = 12 x\left( x^2-\sqrt{\frac{66}{12}}\right) ^2+\left( 2\sqrt{12\cdot 66}-56\right) x\ge 0$ for all $x\ge 1$.

### A.3 Theorem 3

For (4.20), after rescaling $r=Mx$, it suffices to prove that

$$\begin{aligned} p(x)&=2 x^{10}+19 x^9-43 x^8-312 x^7+1056 x^6+712 x^5-8600 x^4\\  &\quad +17920 x^3-17920 x^2+8960 x-1792\ge 2 \end{aligned}$$

holds for $x\ge 1$. In view of $p(1)=2$, $p^\prime (1)=39$ and $p^{\prime \prime }(1)=436$, this clearly follows if we can establish $p^{(3)}(x) \ge 0$ for all $x \ge 1$, as then *p* itself is monotonically increasing for $x\ge 1$.

To show $p^{(3)}(x) \ge 0$ for all $x \ge 1$, we will show that

- $p^{(3)}$ has a unique absolute minimum in $[1,\infty )$ lying in the interval $[1.067,1.068] \subset [1,\infty )$,
- $p^{(3)}$ is strictly positive in all of [1.067, 1.068] hence in all of $[1,\infty )$.

The first part follows from $p^{(4)}(1)=-7584$, $p^{(4)}(\infty )=\infty$ and $p^{(5)}(x)\ge 0$ for all $x \ge 1$ (which we verify immediately below) as well as computing $p^{(4)}(1.067)<0$ and $p^{(4)}(1.068)>0$. The second part follows from estimating negative summands in the polynomial by evaluating them at 1.068 and positive summands at 1.067. It remains to show $p^{(5)}(x)\ge 0$ for all $x \ge 1$. To do this, we will show that

- $p^{(5)}$ has a unique absolute minimum in $[1,\infty )$ lying in the interval $[1.1192,1.11921] \subset [1,\infty )$,
- $p^{(5)}$ is strictly positive in all of [1.1192, 1.11921] hence in all of $[1,\infty )$.

This is established analogously to the previous paragraph noting that obviously

$$ p^{(7)} (x) = 20160\left( 60x^3 +171x^2-86x -78\right) \ge 0 \text { for all } x \ge 1. $$

We turn to (4.22) where after again rescaling $r=Mx$ we need to establish positivity of

$$\begin{aligned} q(x)&= 64 - 1216 x + 4640 x^2 - 7200 x^3 + 4788 x^4 - 732 x^5 - 444 x^6 \\  &\quad + 92 x^7 + 4 x^8 + 4 x^9 + 3 x^{10} \ge 0 \ \ \ \text {for all }x \ge 1. \end{aligned}$$

To do so, we will show that *q* has a unique (absolute) minimum in $[1,\infty )$ and that *q* is positive there.

We note $q^{(5)}(x) =-87840 - 319680 x + 231840 x^2 + 26880 x^3 + 60480 x^4 + 90720 x^5 \ge -87840 + 90240x >0$ for $x\ge 1$. Since $q^{(4)}(1)=-21552<0$ and $q^{(4)}(\infty )=\infty$ it follows that $q^{(4)}$ has a single zero in $[1,\infty )$, corresponding to an absolute minimum of $q^{(3)}(x)$ in $[1,\infty )$. Since also $q^{(3)}(1)<0$ and $q^{(3)}(\infty )=\infty$, we conclude that $q^{(3)}$ has a single zero in $[1,\infty )$, which corresponds to an absolute minimum of $q^{(2)}$ in $[1,\infty )$. Since $q^{(2)}(1)=222$, $q^{(2)}(1.3)<0$, $q^{(2)}(\infty )=\infty$ we have that $q^{(2)}$ has two zeros in $[1,\infty )$ corresponding to a local maximum of $q^\prime$ in [1, 1.3] and a local minimum of $q^\prime$ in $[1.3,\infty )$. Since $q^\prime (1)=34$ we conclude that $q^\prime$ has at most two zeros in $[1,\infty )$ and hence at most one where $q^\prime$ changes sign from + to − corresponding to a minimum of *q*. Since $q^\prime (1.54931)<0$ and $q^\prime (1.54933)>0$ we conclude that *q* itself has an absolute minimum in [1.54931, 54933]. Estimating the negative summands of the polynomial at $x= 1.54933$ and the positive ones at $x=1.54931$ we conclude $q>0$ in [1.54931, 54933] and hence in $[1,\infty )$.

### A.4 Theorem 4

After rescaling $r=2Mx$ in (4.27) we need to establish

A.1 $$\begin{aligned} p(x)&:= 8(x+3) q(x) := 8 (x+3) (-810 + 3159 x - 648 x^2 - 696 x^3 \nonumber \\  &\quad + 144 x^4 + 32 x^5) > 0 \ \ \ \text {for }x\ge 1. \end{aligned}$$

In view of $q(1)>0$ and $q(\infty )=\infty$ it suffices to show $q(x)>0$ at all of its local minima in $[1,\infty )$ to conclude $p(x) \ge 0$ in $[1,\infty )$. Clearly $q^{(3)}(x) =-4176 + 3456 x + 1920 x^2\ge 0$ for $x\ge 1$, which using $q^{\prime \prime }(1)<0$ and $q^{\prime \prime }(\infty )=\infty$ implies that $q^{\prime \prime }$ has a single zero in $[1,\infty )$ corresponding to a unique minimum of $q^\prime$. It follows that $q^\prime$ can have at most two zeros, and at most one where it changes sign from − to + corresponding to a minimum of *q*. We now check that $q^\prime (2.17)<0$ and $q^\prime (2.2)>0$ and that $q(x) >0$ in [2.17, 2.2] (hence in $[1,\infty )$) by estimating the positive summands of *q* at $x=2.17$ and the negative ones at 2.2.

## Footnotes

1 A fixed point $q$ of a flow generated by a vector field $F\,:U\subset \mathbb {R}^2\rightarrow \mathbb {R}^2$ is *hyperbolic*, if the Jacobian matrix $D_qF$ has no eigenvalues with zero real parts.

## References

[1] Andersson, L., Blue, P.: Hidden symmetries and decay for the wave equation on the Kerr spacetime. Ann. Math. (2) **182**(3), 787–853 (2015)

[2] Angelopoulos, Y., Aretakis, S., Gajic, D.: Late-time asymptotics for the wave equation on spherically symmetric, stationary spacetimes. Adv. Math. **323**, 529–621 (2018)

[3] Angelopoulos, Y., Aretakis, S., Gajic, D.: Late-time asymptotics for the wave equation on extremal Reissner–Nordström backgrounds. Adv. Math. **375**, 107363 (2020) 10.1007/s00220-020-03857-3 PMC7591141 33132402

[4] Angelopoulos, Y., Aretakis, S., Gajic, D.: Late-time tails and mode coupling of linear waves on Kerr spacetimes. Adv. Math. **417**, 108939 (2023)

[5] Angelopoulos, Y., Aretakis, S., Gajic, D.: Price’s law and precise late-time asymptotics for subextremal Reissner–Nordström black holes. Ann. Henri Poincaré **24**(9), 3215–3287 (2023)

[6] Aretakis, S.: Stability and instability of extreme Reissner–Nordström black hole spacetimes for linear scalar perturbations I. Commun. Math. Phys. **307**(1), 17–63 (2011)

[7] Blue, P., Soffer, A.: Semilinear wave equations on the Schwarzschild manifold. I. Local decay estimates. Adv. Differ. Equ. **8**(5), 595–614 (2003)

[8] Burq, N., Guillarmou, C., Hassell, A.: Strichartz estimates without loss on manifolds with hyperbolic trapped geodesics. Geom. Funct. Anal. **20**(3), 627–656 (2010)

[9] Cardoso, V., Costa, J.L., Destounis, K., Hintz, P., Jansen, A.: Strong cosmic censorship in charged black-hole spacetimes: still subtle. Phys. Rev. D **98**(10), 104007 (2018) 10.1103/PhysRevLett.120.031103 29400502

[10] Dafermos, M., Rodnianski, I.: A note on energy currents and decay for the wave equation on a Schwarzschild background (2007). arXiv:0710.0171

[11] Dafermos, M., Rodnianski, I.: The wave equation on Schwarzschild–de Sitter spacetimes (2007). arXiv:0709.2766

[12] Dafermos, M., Rodnianski, I.: The red-shift effect and radiation decay on black hole spacetimes. Commun. Pure Appl. Math. **62**(7), 859–919 (2009)

[13] Dafermos, M., Rodnianski, I.: A new physical-space approach to decay for the wave equation with applications to black hole spacetimes. In: XVIth International Congress on Mathematical Physics, pp. 421–432. World Sci. Publ., Hackensack (2010)

[14] Dafermos, M., Rodnianski, I., Shlapentokh-Rothman, Y.: Decay for solutions of the wave equation on Kerr exterior spacetimes III: the full subextremal case . Ann. Math. (2) **183**(3), 787–913 (2016)

[15] Dafermos, M., Rodnianski, I., Shlapentokh-Rothman, Y.: A scattering theory for the wave equation on Kerr black hole exteriors. Ann. Sci. Éc. Norm. Supér. (4) **51**(2), 371–486 (2018)

[16] Dafermos, M., Holzegel, G., Rodnianski, I., Taylor, M.: Quasilinear wave equations on asymptotically flat spacetimes with applications to Kerr black holes. Anal. PDE (2022). arXiv:2212.14093

[17] Dyatlov, S.: Quasi-normal modes and exponential energy decay for the Kerr-de Sitter black hole. Commun. Math. Phys. **306**(1), 119–163 (2011)

[18] Dyatlov, S.: Asymptotics of linear waves and resonances with applications to black holes. Commun. Math. Phys. **335**(3), 1445–1485 (2015)

[19] Dyatlov, S.: Spectral gaps for normally hyperbolic trapping. Ann. Inst. Fourier (Grenoble) **66**(1), 55–82 (2016)

[20] Dyatlov, S., Zworski, M.: Trapping of waves and null geodesics for rotating black holes. Phys. Rev. D **88**, 084037 (2013)

[21] Giorgi, E.: The linear stability of Reissner–Nordström spacetime: the full subextremal range . Commun. Math. Phys. **380**(3), 1313–1360 (2020)

[22] Hintz, P.: A sharp version of Price’s law for wave decay on asymptotically flat spacetimes. Commun. Math. Phys. **389**(1), 491–542 (2022)

[23] Hirsch, M.W., Pugh, C.C., Shub, M.: Invariant Manifolds. Lecture Notes in Mathematics, vol. 583. Springer, Berlin–New York (1977)

[24] Holzegel, G., Kauffman, C.: The wave equation on subextremal Kerr spacetimes with small non-decaying first order terms (2023). arXiv:2302.06387

[25] Holzegel, G., Kauffman, C.: The wave equation on the Schwarzschild spacetime with small non-decaying first-order terms. J. Hyperbolic Differ. Equ. **20**(4), 825–834 (2023)

[26] Katok, A., Hasselblatt, B.: Introduction to the Modern Theory of Dynamical Systems. Encyclopedia of Mathematics and its Applications, vol. 54. Cambridge University Press, Cambridge (1997)

[27] Laul, P., Metcalfe, J.: Localized energy estimates for wave equations on high-dimensional Schwarzschild space-times. Proc. Am. Math. Soc. **140**(9), 3247–3262 (2012)

[28] Marzuola, J., Metcalfe, J., Tataru, D., Tohaneanu, M.: Strichartz estimates on Schwarzschild black hole backgrounds. Commun. Math. Phys. **293**(1), 37–83 (2010)

[29] Mavrogiannis, G.: Morawetz estimates without relative degeneration and exponential decay on Schwarzschild–de Sitter spacetimes. Ann. Henri Poincaré **24**(9), 3113–3152 (2023)

[30] Mavrogiannis, G.: Relatively non-degenerate integrated decay estimates on subextremal Kerr–de Sitter (2025). arXiv:2503.22090

[31] Nicolas, J.-P.: Conformal scattering on the Schwarzschild metric. Ann. Inst. Fourier **66**(3), 1175–1216 (2016)

[32] Schlue, V.: Decay of linear waves on higher-dimensional Schwarzschild black holes. Anal. PDE **6**(3), 515–600 (2013)

[33] Shlapentokh-Rothman, Y., da Costa, R.T.: Boundedness and decay for the Teukolsky equation on Kerr in the full subextremal range : physical space analysis (2023). arXiv:2005.13644 10.1007/s00205-026-02173-9 PMC13498544 42632954

[34] Stogin, J.: Nonlinear Wave Dynamics in Black Hole Spacetimes. Ph.D. thesis. Princeton University (2017). https://dataspace.princeton.edu/handle/88435/dsp01p5547t983

[35] Tataru, D., Tohaneanu, M.: A local energy estimate on Kerr black hole backgrounds. Int. Math. Res. Not. IMRN **2**, 248–292 (2011)

[36] Vasy, A.: Microlocal analysis of asymptotically hyperbolic and Kerr-de Sitter spaces (with an appendix by Semyon Dyatlov). Invent. Math. **194**(2), 381–513 (2013)

[37] Wunsch, J., Zworski, M.: Resolvent estimates for normally hyperbolic trapped sets. Ann. Henri Poincaré **12**(7), 1349–1385 (2011)

[38] Zehnder, E.: Lectures on Dynamical Systems. EMS Textbooks in Mathematics. European Mathematical Society (EMS), Zürich (2010)

